# Lack of NHE6 and Inhibition of NKCC1 Associated With Increased Permeability in Blood Labyrinth Barrier-Derived Endothelial Cell Layer

**DOI:** 10.3389/fncel.2022.862119

**Published:** 2022-04-12

**Authors:** Marijana Sekulic-Jablanovic, Jessica Paproth, Cinzia Sgambato, Giuseppe Albano, Daniel G. Fuster, Daniel Bodmer, Vesna Petkovic

**Affiliations:** ^1^Department of Biomedicine, University Hospital Basel, University of Basel, Basel, Switzerland; ^2^Inselspital Bern, Department of Biomedical Research, University of Bern, Bern, Switzerland; ^3^Clinic for Otolaryngology, Head and Neck Surgery, University Hospital Basel, Basel, Switzerland

**Keywords:** blood-labyrinth barrier, cochlea, NHE6/SLC9A6, hearing loss, NKCC1

## Abstract

Acoustic trauma, autoimmune inner ear disease, and presbycusis feature loss of the integrity of the blood-labyrinth barrier (BLB). Normal BLB function depends on endothelial structural integrity, which is supported and maintained by tight junctions and adherens junctions within the microvascular endothelial layer. When these junctions are disrupted, vascular leakage occurs. Tight junctions and adherens junctions are functionally and structurally linked, but the exact signaling pathways underlying their interaction remain unknown. In addition, solute carriers (SC) are essential for optimal exchange through BLB. Previously, we found that SC family member, the sodium–hydrogen exchanger NHE6, was expressed in all wildtype cochlear tissues, and that *Nhe6*-knockout mice displayed moderate hearing loss. Moreover, NHE6 depletion affected Trk protein turnover and endosomal signaling. Here, we investigated whether NHE6 might impact BLB integrity. We found that *Nhe6*-knockout, BLB-derived endothelial cells showed reduced expression of major junctional genes: *Tjp1*, *F11r*, *Ocln*, *Cdh5*, and *Cldn5*. Co-culturing BLB-derived endothelial cells with pericytes and/or perivascular resident macrophage-like melanocytes in a transwell system showed that monolayers of *Nhe6*-knockout BLB-derived cells had lower electrical resistance and higher permeability, compared to wildtype endothelial monolayers. Additionally, another SC, NKCC1, which was previously linked to congenital deafness, was downregulated in our *Nhe6*-knockout mouse model. Blocking NKCC1 with a NKCC1-specific inhibitor, bumetanide, in wildtype BLB-derived endothelial cells also caused the downregulation of major junctional proteins, particularly *Tjp1* and *F11r*, which encode the zonula occludens and junctional adhesion molecule-1 proteins, respectively. Moreover, bumetanide treatment increased cell permeability. In conclusion, we showed that the lack or inhibition of NHE6 or NKCC1 affected the permeability of endothelial BLB-derived cells. These findings suggested that NHE6 and NKCC1 could serve as potential targets for modifying BLB permeability to facilitate drug delivery across the BLB to the cochlea or to protect the cochlea from ototoxic insults.

## Introduction

Normal hearing depends on the proper function of the stria vascularis (SV) and spiral ligament (SL), located in the lateral wall of the cochlear duct. The SV is responsible for producing the endocochlear electrical potential and maintaining the ion composition of the endolymph (Tasaki and Spyropoulos, 1959). The blood-labyrinth barrier (BLB) within the cochlear SV and SL is a highly specialized capillary network that controls exchanges between blood and the intrastiral space. It enables the selective passage of ions, fluids, and nutrients, maintains cochlear homeostasis, and restricts the influx of blood-born toxic substances (Wangemann, 2006). The BLB comprises capillary endothelial cells, well-developed tight junctions (TJs) and adherens junctions (AJs), pericytes, basement membrane, and perivascular resident macrophage-like melanocytes (PVM/Ms). Interactions and signaling between the endothelial cells, pericytes, and PVM/Ms maintain the fine-tuned regulation of vascular permeability and preserve hearing integrity. Inflammation, noise trauma, aging, or mutations can disrupt the BLB integrity; this increases vascular permeability and causes cochlear edema, which is associated with a number of hearing disorders (Lin and Trune, 1997; Neng et al., 2015; Zhang et al., 2015).

Previously, we investigated the role of solute carrier (SC) family member, *Slc9a6*, which encodes the sodium–hydrogen exchanger 6 (NHE6) within the mouse cochlea, and we focused mainly on the organ of Corti. Sodium–hydrogen exchangers are membrane transporters that exchange Na^+^ ions for protons, which regulates the pH within the cell or cellular compartment. NHE6 controls the pH of endosomes, which is important for endosome recycling. In addition, NHE6 may contribute to protein trafficking. We showed that *Nhe6*-knockout mice displayed significant hearing loss, compared to wildtype littermates, based on elevated hearing thresholds in click-induced and frequency-specific auditory brainstem response measurements. We found that auditory stimuli above the elevated threshold could travel normally along the successive nuclei in the central auditory pathway. That finding indicated that hearing loss in *Nhe6*-knockout animals was due to cochlear damage, and that NHE6 was important for normal hearing function. Those results were based on experiments involving sensory structures in the organ of Corti.

Currently, the physiology and overall properties of the cochlear BLB remain incompletely elucidated, despite the importance of the BLB in transporting substances between the blood and intrastrial space. After isolating endothelial cells, pericytes, and PVM/Ms, we found that a co-culture of all three cell types had the best integrity, and that *Nhe6*-knockout exhibited greater permeability than wildtype. Accordingly, we reasoned that, in addition to its role in the organ of Corti, *Nhe6* may play an important role in the integrity of the BLB in the SV (Kucharava et al., 2020).

The exact mechanisms that regulate the BLB permeability are not well-understood. BLB microvascular endothelial cells are connected by AJs and TJs, and they are supported by the basement membrane, pericytes, and PVM/Ms. It was found that pericytes and PVM/Ms regulated TJ gene expression in endothelial cell monolayers, contributing to greater endothelial cell monolayer integrity and reduced permeability (Neng et al., 2013).

In the present study, we investigated the mechanism underlying the hearing impairment in *Nhe6*-knockout mice by analyzing BLB integrity. We performed RNAseq analyses of primary endothelial cell cultures and found that *Cadh5* was among the 30 most downregulated genes. This prompted us to screen several additional AJ- and TJ-related genes (*Tjp1*, *F11r*, *Occl*, *Cadh5*, and *Cldn*) with quantitative PCR to compare expression levels between knockout and wildtype cells. We found that each of the tested genes was downregulated to a different extent in knockout cells, and lower expression corresponded to higher permeability in the BLB. In addition, we investigated whether blocking another solute carrier, NKCC1 (encoded by the *Slc12a2* gene), Cl^–^, Na^+^, and/or K^+^ ion transporter involved in hearing impairments, might also affect AJ and TJ gene expression in a wildtype-derived endothelial cell monolayer (Gamba, 2005).

## Materials and Methods

### Animal Procedures

All animal procedures were conducted in compliance with the European Communities Council Directive of 24 November, 1986 (86/609/EEC), and they were approved by the Kantonales Veterinäramt, Basel, Switzerland. Steven Walkley of Albert Einstein College of Medicine (Bronx, NY) provided animals with a targeted Nhe6 gene disruption (Jackson Laboratories Stock# 005843, strain name B6.129P2-Slc9a65tm1Dgen). All mice described here were backcrossed for >10 generations into a C57bl6/j (WT) background. Cochleae for culture studies were obtained from mice sacrificed by decapitation on postnatal day 10–12. Animals were maintained on a 12-h light/12-h dark schedule with free access to water and a standard mouse diet.

### Primary Cell Culture Isolation

We generated three primary cell lines with the same protocol, then each cell type was cultured in the appropriate medium. Briefly, cochleae from 10 to 12 days-old mice were harvested under sterile conditions. The SV with SL was gently pulled out and placed in ice-cold PBS. The isolated tissue was cut into small pieces and transferred to a clean 35-mm dish. Tissue fragments were trypsinized with 0.05% trypsin/EDTA for 5 min at 37°C; the reaction was blocked with blocking solution [DMEM and 10% fetal bovine serum (FBS)]. Next, the mixture was pipetted several times to break down the tissue, followed by centrifugation for 5 min at 500 rpm. Then, cells were seeded in 6-well collagen-coated plates (Falcon, cat#353046) at a roughly uniform density, and incubated at 37°C in 5% CO_2_. The cells rested for 48 h, then the medium was replaced every 3 days. Once cells reached higher confluency, the medium was replaced every 2 days. Cell clusters of each phenotype formed at 6–7 days post isolation; then, cells were passaged into 25-cm^2^ collagen-coated flasks (Thermo Scientific, United States, cat#156367). Cultured cells were purified with fluorescence-activated cell sorting according to protocol from Zhang et al. (2017), collected in culture media and cultured in 25 mm^2^ collagen-coated flasks and used for subsequent experiments.

### Culturing Media and Cell Treatment

Endothelial cells were cultured in Endothelial Cell Basal Medium (Sigma, United States, cat#210-500) with 10% FBS (Gibco, United States, cat#10270106), 1% endothelial cell growth factor (Sigma, United States, cat#E1388), and 1% penicillin/streptomycin solution (Sigma, United States, cat#P4333). Pericytes were cultured in Dulbecco’s modified Eagle’s medium (Gibco, United States, cat#12491023) containing 10% FBS (Gibco, United States, cat#10270106), 100 nM pigment epithelium-derived factor (Sigma, United States, cat# SRP4988), and 1% penicillin/streptomycin solution (Sigma, United States, cat#P4333). PVM/Ms were cultured in 254CF medium (Thermo Scientific, United States, cat#M254CF500) containing 1% human melanocyte growth supplement (Gibco, United States, cat#S0025), 10% FBS (Gibco, United States, cat#12491023), and 0.2% gentamicin/amphotericin B solution (Gibco, United States, cat#R01510). For treatment experiments, confluent wildtype BLB-derived endothelial cells were treated with 30 μM bumetanide for 48 h.

### Western Blotting

Endothelial cells were homogenized in cell lysis buffer (Sigma–Aldrich, St. Louis, MO, United States, cat#C3228) with a protease inhibitor cocktail (Sigma–Aldrich, St. Louis, MO, United States, cat#C3228, Cat#P8340) for 1 min on ice. Protein concentrations were determined by the Bradford method using Bio-Rad Protein Assay Dye Reagent Concentrate (Bio-Rad, Switzerland, cat#5000006). Protein concentrations were confirmed using a NanoDrop (Thermo Fisher Scientific, Waltham, MA, United States). Samples were mixed with Laemmli sample buffer (Sigma–Aldrich, St. Louis, MO, United States, cat#S3401), heated at 95°C for 5 min, and then resolved on 4–20% Mini-PROTEAN ^®^ TGX™ precast protein gels (Bio-Rad, Switzerland, cat#4561096). Each endothelial cell culture was obtained from three mice per phenotype and experiment and individual experiments performed three times. After electrophoresis, separated proteins were transferred onto polyvinylidene fluoride membranes. Membranes were first incubated with 5% non-fat dry milk dissolved in PBS-T for 1 h at room temperature to block non-specific protein binding sites. Next, the membranes were washed with PBS-T (3 × 10 min) and then incubated overnight at 4°C in 5% non-fat dry milk in PBS with one of the following primary antibodies: rabbit anti-VE-cadherin (Thermo Fisher, United States, cat#PA5-19612), rabbit anti-NKCC1 (Thermo Fisher, United States, cat# PA5-98154). The membranes were then washed with PBS-T (3 × 10 min) and incubated for 1 h at room temperature with the appropriate horseradish peroxidase-conjugated secondary antibody. After washing, immunoreactive protein bands were visualized using Super Signal West Dura Extended Duration Substrate (Thermo Fisher Scientific, Waltham, MA, United States, Cat#34076). An anti-β-actin antibody was used as a control to demonstrate equivalent protein loading. The intensity of the immuno-positive bands was determined using Fiji-win 32 software, capturing the identical regions on each blot, deducting the background signal. The intensity of all proteins of interest was normalized to the intensity of β-actin in the same sample.

### Immunofluorescence

Test samples of each cell phenotype were grown on 4-well collagen-coated glass-bottom dishes (Ibidi, Germany, cat#80426). Cells were fixed in 4% paraformaldehyde (Sigma, United States, cat# 158127) in PBS (Sigma, United States, cat# P4417), permeabilized with 0.1% Triton X-100 (Sigma, United States, cat#X100) in PBS, then incubated for 1 h at room temperature with anti-von Willebrand factor (vWf, Sigma, United States, cat#F3520), anti-PDGFR-β (Santa Cruz Biotechnology, United States, cat#sc374573), anti-F4/80 (Abcam, United Kingdom, cat#ab6640), anti-ZO1 or anti-NKCC1 (Thermo Fisher, United States, cat#33-9100, cat#PA5-66620) primary antibodies. Next, samples were washed and incubated with appropriate secondary antibodies (Thermo Fisher, United States, cat#A2124, cat#A-11001, cat#A-11008) as well as Alexa Fluor 488- or Rhodamine-labeled phalloidin (Thermo Fisher, United States, cat#A12379, cat#R415) in PBS-T for 1 h at room temperature. Samples were washed with PBS and incubated with DAPI for 5 min. Then, cells were washed with PBS and mounted on microscope slides with Dako Fluorescent Mounting medium (Dako, Denmark, cat#S3023). Images were captured by Nikon Eclipse Ti2 inverted widefield microscope. The intensity of the immunofluorescence signal was determined using Fiji-win 32 software.

### Mono-Culture and Co-culture Models

For the mono-culture, purified endothelial cells were passaged 3 times, seeded at a density of 4 × 10^4^/cm^2^, and grown on Transwell membrane inserts (Corning, United States, cat#3470) coated with collagen. For cultures, pericytes, with or without PVM/Ms, were combined with endothelial cells (harvested after three passages and seeded at a density of 4 × 10^4^/cm^2^), then grown on the fibronectin coated inserts for at least 5 days. Pericytes and PVM/Ms were used between passages 3 and 5. Cells were passaged at 80–90% confluency. Inserts contained 800 ml of medium in the basolateral compartment and 250 μl in the apical compartment.

### Transepithelial Electrical Resistance and Endothelial Monolayer Permeability Assessment

Transepithelial electrical resistance (TEER) was measured with an Epithelial Voltohmmeter (EVOM). We used an AC square wave at a frequency of 12.5 Hz to avoid any charging effects on the electrodes or the cell layer. The EVOM measurement range was 1–9999 Ω, and the resolution was 1 Ω. The EVOM included a pair of electrodes, popularly known as STX2 or “chopstick” electrodes. Each stick of the electrode pair (4-mm wide and 1-mm thick) contained a silver/silver chloride pellet for measuring voltage and a silver electrode for passing current. The measurement procedure included measuring the blank resistance (R_BLANK_) of the semipermeable membrane only (without cells), and then measuring the resistance across the cell layer on the semipermeable membrane (R_TOTAL_). The cell specific resistance (R_TISSUE_) in units of Ω, can be obtained as:


$${\text{R}}_{\text{TISSUE}}\,\left(\Omega \right)={\text{R}}_{\text{TOTAL}}-{\text{R}}_{\text{BLANK}}$$

Where resistance is inversely proportional to the effective area of the semipermeable membrane (M_AREA_) which is reported in units of cm^2^.


$${\text{R}}_{\text{TISSUE}}\,\left(\Omega \right)\,\alpha \,\frac{1}{{\text{M}}_{\text{AREA}}\,\left({\text{cm}}^{2}\right)}$$

TEER value is reported in units of Ω′⋅cm2 and calculated as:


$${\text{TEER}}_{\text{REPORTED}}={\text{R}}_{\text{TISSUE}}\,\left(\Omega \right)\times {\text{M}}_{\text{AREA}}\,\left({\text{cm}}^{2}\right)$$

After cultures reached steady-state TEER, we measured the permeability of the endothelial cell monolayer to FITC-dextran (MW: 70 kDa). We measured three types of cell monolayers: an endothelial cell monolayer, a co-culture monolayer, and a tri-culture monolayer. The apparent permeability coefficient (Papp) of dextran was determined, as follows: Papp = Δ*Q*(t)/*Ac*_0_Δt, where Δ*Q*/Δ*t* is the permeation rate (μM s^–1^) of the dextran across the microporous membrane, which is calculated from the initial straight slopes; *A* is the surface area of the membrane (i.e., 0.32 cm^2^), and *c*_0_ is the initial dextran concentration in the donor chamber at *t* = 0 (μM) (Artursson, 1990). Experiments were performed in triplicate, and results are presented as the mean ± SD.

### RNA Sequencing

The RNA isolation, preparation, quality assessment, and sequencing and the differential gene expression analyses were performed by Microsynth AG, which had a certified quality management system (ISO 9001:2015). New generation sequencing and Sanger sequencing were accredited according to the standard ISO/IEC 17025:2005 (STS 0429). Briefly, we isolated cells of each phenotype from a minimum of six mice per phenotype and culture. Three culture samples for each phenotype were sent for analysis. The cells were dissociated in RNA lysis buffer and sent to Microsynth AG on dry ice. The bioinformatic analysis included a standard eukaryotic differential gene expression analysis (reference mm10). The sequencing included 220,745,743 total past filter reads, 16,445,368,036 total past filter bases, and a mean read length of 75 bp.

### Quantitative PCR Protocol and RNA Isolation

RNA was isolated from 10 SV and SL per phenotype and experiment with a Direct-Zol RNA MiniPrep kit (Zymo Research, Germany, cat#R2050), according to the manufacturer’s instructions. Total RNA (1000 ng) was reverse transcribed with a High-Capacity cDNA Reverse Transcription Kit (Applied Biosystems, United States). We analyzed triplicate samples with quantitative PCR on an ABI Prism 7900HT Sequence Detection System (Applied Biosystems, United States), with the Power Sybr Green Master Mix (Applied Biosystems, United States). Primers (synthesized by Microsynth, St. Gallen, Switzerland) that targeted *vWf*, *Slc2a1*, *Cd34*, *Des*, *Pdgfrb*, *Cspg4*, *Gsta4*, *Emr1*, *Tjp1*, *F11r*, *Ocln*, *Cdh5*, *Cldn5*, and *Gapdh* were added at a final concentration of 250 nM per reaction. The primer sequences are listed in Supplementary Table 1. The relative quantities of specifically amplified cDNAs were calculated with the comparative threshold cycle method, and *Gapdh* expression levels were used as the endogenous reference.

### Statistical Analysis

Statistical analyses were performed with GraphPad Prism software (San Diego, CA, United States). Multiple groups were compared with a two-way analysis of variance, and two groups were compared with an unpaired Student’s *t*-test. Data were confirmed to be normally distributed by conducting a Shapiro-Wilk test. *P*-values < 0.05 were considered significant.

## Results

### *Nhe6*-Knockout Downregulated Adherens and Tight Junction Gene Expression

**FIGURE 1:**
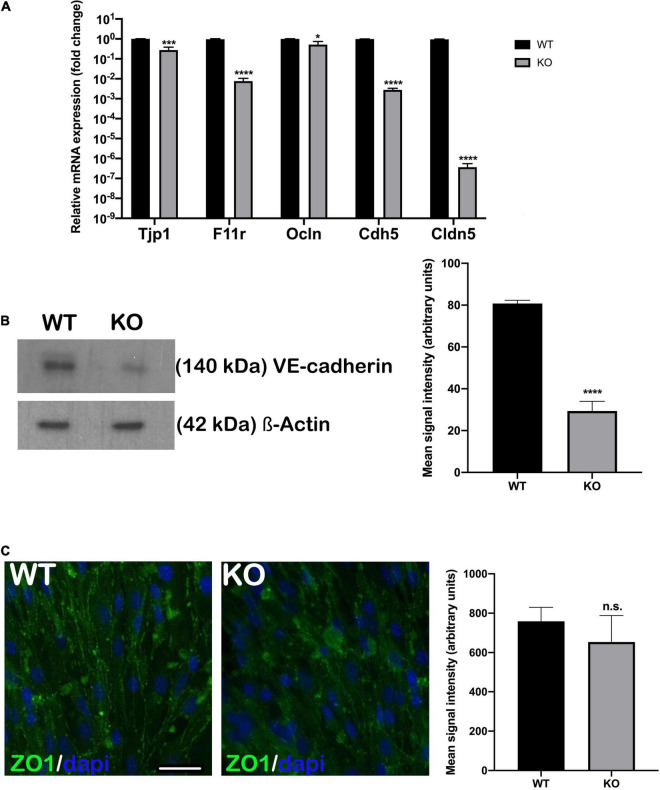
Endothelial junctional gene expression was downregulated in *Nhe6*-knockout cells. **(A)** Relative expression of genes that encode the major adherens and tight junction proteins, *Tjp1*, *F11r*, *Ocln*, *Cdh5*, and *Cldn5* are compared between *Nhe6*-knockout (KO) and wildtype (WT) cells. The overall expression levels of adherens and tight junction-related genes were significantly lower in KO than in WT cells, and the most prominent downregulations were in *Tjp1*, *Cdh5*, and *Cldn5*. Data represent the mean (SD); (*n* = 3). **(B, left panel)** Representative western blot image showing decreased VE-cadherin protein presence in *Nhe6*-knockout EC cell lysate. **(B, right panel)** Western bolt signal quantification. Each endothelial cell culture was obtained from three mice per phenotype and experiment, and individual experiments performed three times (*n* = 3); Data represent the mean (SD); *P < 0.05, ***P < 0.001, ****P < 0.0001. ß-actin was used as a loading control. **(C, left panel)** Immunostaining shows the expression of tight junction protein, ZO1 (green), in WT and KO endothelial cells. **(C, Right panel)** ZO1 immunofluorescence signal quantification. Samples from five mice were pooled per phenotype and experiment; (*n* = 3); Scale bar, 50 μm. Data represent the mean (SD); (*n* = 3); n.s., not significant.

The permeability of endothelial monolayers relies on the abundance and proper formation of cell-cell junctions. After establishing appropriate selective culture conditions and confirming each primary cell type phenotype (Supplementary Figure 1), we first evaluated the expression of major genes involved in forming adherens and tight junctions in endothelial cell monolayers: *Tjp1*, *F11r*, *Ocln*, *Cdh5*, and *Cldn5*. We compared gene expression levels between the *Nhe6*-knockout and wildtype cells. Overall, the expression levels of adherens and tight junction-related genes were significantly lower in the *Nhe6*-knockout than in wildtype cells, and the reduced levels were observed for *Tjp1*, *F11r*, *Cdh5*, *Ocln*, and *Cldn5* (Figure 1A). Low *Cdh5* expression level was confirmed by significantly decreased VE-cadherin protein presence in *Nhe6*-knockout (Figure 1B). In contrast to *Tjp1* gene expression, ZO-1 immunostaining showed no differences in the tight junction protein expression between wildtype and *Nhe6*-knockout cells (Figure 1C).

### Transepithelial Electrical Resistance and Permeability Altered in *Nhe6*-Knockout Compared to Wildtype Cells

**FIGURE 2:**
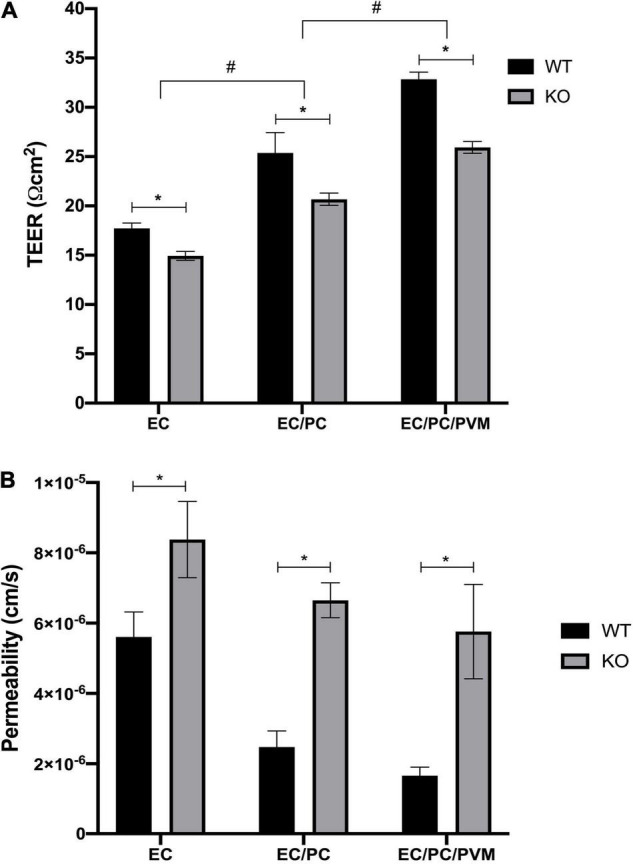
Effects of *Nhe6*-knockout on TEER and permeability of endothelial monolayers. TEER measurements for wild-type (WT) and *Nhe6*-knockout (KO) mice. **(A)** Transwell model drawing with TEER results. Measurements were performed in monolayers of mono-cultures of endothelial cells (EC; light gray columns), co-cultures of ECs and pericytes (EC/PC; medium gray columns), and tri-cultures of EC/PCs and perivascular resident macrophage-like melanocytes (EC/PC/PVM/M; dark gray columns). Error bars represent the mean ± SD; (*n* = 3). *P < 0.05; significant difference between WT and KO in each culturing condition. #*P* < 0.05; significant differences between mono-cultures, co-cultures, and tri-cultures. **(B)** Permeability of dextran (70 kDa) across endothelial monolayers, co-culture monolayers, and tri-culture monolayers of WT and KO cells. Dextran was added to the apical chambers of Transwells, at a final concentration of 10 μM. Dextran that permeated to the basolateral chambers was evaluated with the HPLC method. Data represent the mean ± SD; (*n* = 3). *P < 0.05, compared between WT and KO, based on one-way analysis of variance.

Initial TEER measurements showed higher TEER values in monolayers of endothelial cells and pericytes, and particularly, in monolayers of endothelial cells, pericytes and PVM/Ms, compared to an endothelial cell monolayer, in both *Nhe6*-knockout and wildtype samples (Figure 2A). This result was consistent with previous findings that co-cultures of endothelial cells, pericytes, and PVM/Ms contributed to higher membrane integrity, compared to mono-cell cultures (Neng et al., 2013).

Next, we investigated the effects of tight junction protein downregulation in *Nhe6*-knockout cultures by measuring permeability with FITC-labeled 70-kDa dextran. In all three tested culture models, *Nhe6*-knockout monolayers exhibited higher permeability than wildtype monolayers (Figure 2B). This finding prompted us to investigate the whole genome to determine whether any other differences between the two phenotypes might lead to increased permeability in *Nhe6*-knockout monolayers.

### RNA Sequencing Reveals Altered Gene Expression in *Nhe6*-Knockout Cells

To investigate the increased permeability in endothelial cells isolated from *Nhe6*-knockout SVs, we investigated changes in gene expression that might be associated with permeability. RNA sequencing was performed in samples of primary endothelial cell cultures of both *Nhe6*-knockout and wildtype SVs.

**FIGURE 3:**
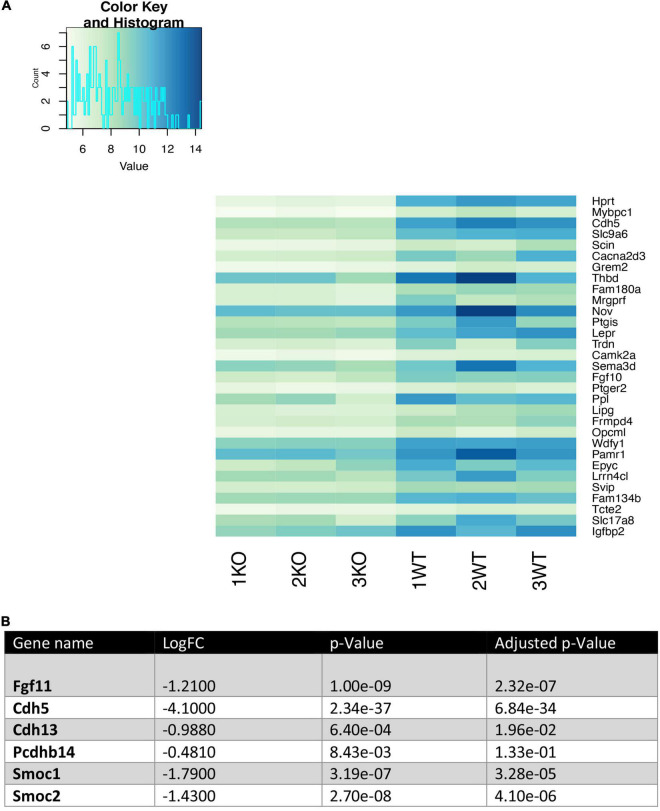
Heatmap of the 30 most downregulated genes in *Nhe6*-knockout compared to wildtype endothelial cells. Heatmap shows RNA sequencing results of the 30 most downregulated genes in *Nhe6*-knockout (KO) compared to wildtype (WT) cells. **(A)** Some of the most prominent differences are evident for *Hprt*, *Cdh5*, *Thbd*, *Nov*, *Ptgis*, *Lepr*, *Fgf10*, *Ppl*, *Wdfy1*, *Epyc*, *Slc17a8*, and *Igfbp2* genes. These genes affect multiple cellular processes, including: cell cohesion, organization of intercellular junctions, barrier stability, cell adhesion, migration, proliferation, differentiation, and survival. **(B)** Several other genes that directly or indirectly influence the integrity of the endothelial cell layer were downregulated in KO compared to WT endothelial cells. Samples from six mice were pooled per phenotype and experiment; (*n* = 3).

We analyzed the 30 most downregulated genes in *Nhe6*-knockout compared to wildtype cells. These genes were involved in various cellular processes. Some of the most prominent differences were found for *Hprt*, *Cdh5*, *Thbd*, *Nov*, *Ptgis*, *Lepr*, *Fgf10*, *Ppl*, *Wdfy1*, *Epyc*, *Slc17a8*, and *Igfbp2* (Figure 3A). *Cdh5* was particularly interesting, because it encodes vascular endothelial cadherin (VE-cadherin), one of the main components of the BLB. VE-cadherin plays the important role of controlling the cohesion and organization of intercellular junctions. Among the other genes of interest, *Thbd* encodes thrombomodulin, an endothelial-specific type I membrane receptor that binds thrombin. Thrombomodulin is involved in anti-inflammatory and barrier-stabilizing processes (Wenzel et al., 2020). *Nov* (nephroblastoma overexpressed) encodes a matricellular protein that regulates multiple cellular activities, including cell adhesion, migration, proliferation, differentiation, and survival (Zhang and Wang, 2011).

In addition, several other genes that influence endothelial cell monolayer integrity were downregulated in *Nhe6*-knockout cells compared to wildtype cells (Figure 3B). For example, *Fgf11* regulated the expression of tight junction proteins in human umbilical vein endothelial cells; indeed, overexpression of *Fgf11* increased the expression of tight junction proteins, occludin, ZO-1, and claudin-5 (Yang et al., 2015). *Cdh13* and *Pcdhb14*, both involved in intercellular connections showed lower expression (Haselton and Heimark, 1997; Bujko et al., 2015). Smoc1 and Smoc2, matricellular proteins found in basement membranes promoting endothelial cell proliferation angiogenesis, matrix assembly and cell adhesiveness were also downregulated in *Nhe6*-knockout (Rocnik et al., 2006; Awwad et al., 2015).

### NKCC1 Inhibition Increases Permeability of Blood-Labyrinth Barrier-Derived Endothelial Cell Monolayers

**FIGURE 4:**
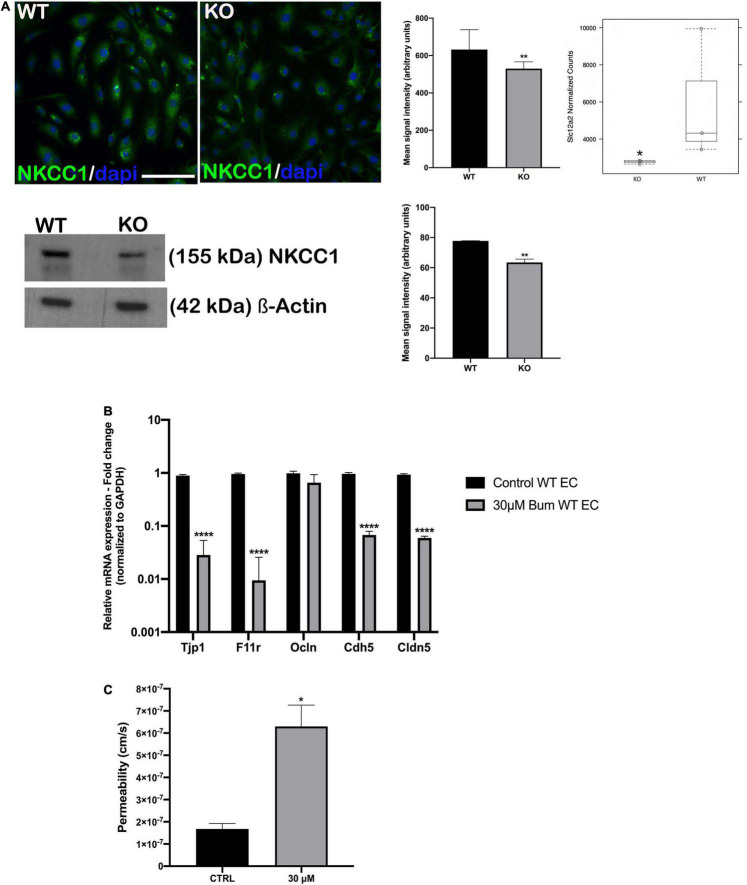
NKCC1 inhibition influences endothelial permeability. **(A, top left panel)** endothelial cells immunostained with anti-NKCC1 antibodies show homogenous protein expression in a primary culture of wildtype (WT) and *Nhe6*-knockout endothelial cells (KO). **(A, middle panel)** NKCC1 immunofluorescence signal quantification. Samples from five mice were pooled per phenotype and experiment; (*n* = 3); Scale bar, 50 μm. Data represent the mean (SD); (*n* = 3); **P < 0.01. **(A, top right panel)** In *Nhe6*-knockout cells (KO), NKCC1 expression was downregulated, compared to WT cells. Data represent the mean ± SD; (*n* = 3); *P < 0.05. **(A, bottom left panel)** Representative western blot image showing decreased NKCC1 protein presence in *Nhe6*-knockout EC cell lysate. **(A, bottom right panel)** western bolt signal quantification. Each endothelial cell culture was obtained from three mice per phenotype and experiment, and individual experiments performed three times (*n* = 3); Data represent the mean (SD); **P < 0.01. **(B)** Relative mRNA expression of the junctional genes after inhibiting NKCC1 with 30 μM bumetanide (bum) in WT endothelial cells (EC). Error bars represent the (SD); (*n* = 3); ****P < 0.0001. **(C)** After inhibiting NKCC1 in WT ECs with 30 μM bumetanide (30 μM), permeability increased compared to untreated control cells (CTRL). Data represent the mean (SD); (*n* = 3). *P < 0.05.

Another SC family member, *Slc12a2*, was downregulated in *Nhe6*-knockout-derived endothelial cells on the gene as well on the protein level (Figure 4A). *Slc12a2* encodes NKCC1, an intrinsic membrane protein that transports chloride, sodium, and/or potassium ions across plasma membranes. Therefore, we investigated whether reduced *Slc12a2* expression alone could contribute to the reduced permeability of *Nhe6*-knockout cell monolayers. We treated confluent wildtype BLB-derived endothelial cells with 30 μM bumetanide, an NKCC1 inhibitor known as loop diuretic, for 48 h (Figure 4B). We found an overall downregulation in all tested junctional genes, including *Tjp1*, *F11r*, *Ocln*, *Cdh5*, and *Cldn5.* The most prominent effects were observed in the expression of *Tjp1* and *F11r*, which encode ZO-1 and JAM1, respectively. The same bumetanide treatment also increased permeability in a transwell model of wildtype endothelial cells co-cultured with pericytes (Figure 4C).

## Discussion

The BLB is crucial in regulating the inner ear ionic homeostasis, transport of fluids and nutrients, and restricting the entry of toxic substances into the inner ear. It possesses an array of enzymes and transporters corresponding to high metabolic demand and robust transport activity (Saito et al., 1997; Yang et al., 2011). As in the brain, various solute carrier transporters are present in BLB managing processes, from the cellular uptake of nutrients to the absorption of drugs and other xenobiotics. Similar to blood–brain barrier (BBB) endothelium of the BLB microvasculature is much tighter than that elsewhere in the body which leads to extremely low overall permeability. Cells associated with BLB endothelium, particularly pericytes and PVM/Ms, appear to be involved in this low permeability (Neng et al., 2013). TJs and AJs are major regulators of vessel permeability. TJs form tight seals between endothelial cells to form a continuous blood vessel wall, and AJs initiate and maintain contact between endothelial cells (Rubin and Staddon, 1999; González-Mariscal et al., 2003). These junctional cells also produce signals that regulate cell growth, apoptosis, and overall vascular homeostasis. Changes in junctional organization could modify the endothelial layer and vessel wall architecture (Dejana, 2004; Gumbiner, 2005).

In the present study, we found that knocking out *Slc9a6* expression in endothelial cells resulted in downregulated expression of *Tjp1*, *F11r*, *Ocln*, *Cdh5*, and *Cldn5* (Figure 1A). The most significant downregulations were in *Cdh5*, *Cldn5*, *and F11r* genes. *Cdh5* encodes VE-cadherin, the major transmembrane protein in the endothelial AJ, and we found a significant decrease in *Nhe6*-knockout derived endothelial cells (Figure 1B). *Tjp1* appeared to be slightly downregulated compared to the three most downregulated genes, and this change was not reflected in ZO1 immunostaining between wildtype and *Nhe6*-knockout (Figure 1C). Claudin and JAM1 proteins are included in the TJ, where they physically associate with their counterparts on the plasma membranes of adjacent endothelial cells (Citi and Cordenonsi, 1998; Martìn-Padura et al., 1998; Rubin and Staddon, 1999; González-Mariscal et al., 2003). Claudins also perform the main barrier function by selectively limiting paracellular ion movement, which produces the high electrical resistance of the barrier (Matter and Balda, 2003; McNeil et al., 2006). Downregulations in claudin-5 and occludin expression were associated with increased BLB permeability in the pathology of noise-induced hearing impairments, although the mechanism remains poorly understood (Wu et al., 2014b). JAMs promote the localization of ZO-1, AF6, CASK, and occludin at points of cell contact (Itoh et al., 2001; Bazzoni and Dejana, 2004).

The BBB TJs contribute to high endothelial electrical resistance and low paracellular permeability and the BLB shows similar properties (Crone and Christensen, 1981; Butt et al., 1990). Followed by decreased expression of the major junctional genes, we investigated functional aspects of the endothelial barrier in *Nhe6*-knockout BLB cell derived transwell model by measuring electrical resistance and permeability. We found that electrical resistance was significantly reduced in *Nhe6*-knockout monolayers, compared to wildtype monolayers, for all mono- and co-culture samples. The most prominent finding was the significant reduction in TEER in the tri-culture model of *Nhe6*-knockout-derived monolayers, compared to wildtype monolayers (Figure 2A). Furthermore, FITC-labeled 70-kDa dextran permeability was significantly increased in monolayers of all three types of *Nhe6*-knockout-derived cultures.

Our results were consistent with previous findings that showed that tri-cultures of endothelial cells, pericytes, and PVM/Ms significantly reduced the permeability of monolayers, compared to endothelial cell monolayers (Neng et al., 2013). In another study, lipopolysaccharides (LPS) were found to induce middle ear inflammation. *In vivo* (animal-based) and *in vitro* (cell line-based) models showed that LPS disrupted BLB integrity by significantly downregulating TJ protein expression, at both the transcript and protein levels. Similar to our findings in *Nhe6*-knockout endothelial cells, they found that LPS reduced the expression levels of VE-cadherin, occludin, and ZO-1 proteins in endothelial cells (Zhang et al., 2015).

Previously, we found that the loss of NHE6 affected hearing. We proposed that this outcome might be due to impaired Trk protein turnover, due to altered endosomal signaling. Many studies in non-neuronal cells have connected endosomal NHE activity with endosomal pH regulation and vesicular traffic control. Endothelial-cell uptake cargos become associated with NHE6-positive compartments within 3 h after endocytosis. Moreover, abolishing Na^+^/H^+^ gradients blocked lysosomal delivery and rerouted traffic to the cell surface (Kondapalli et al., 2014). In addition to their role in trafficking, endosomes serve as platforms where various signaling cascades are being activated or maintained. The endosomal pathway has been shown to play a role in mRNA localization and translation (Baumann et al., 2014). Two key players of the endosomal system are the early and late endosomes, which are associated with Rab5 and Rab7 guanosine triphosphatases, respectively. RNA-bearing Rab7 late endosomes provide sites for local translation. Disruption of Rab7 decreases protein synthesis and mitochondrial integrity (Cioni et al., 2019). In our previous *Nhe6*-knockout study we found no change in Rab5 levels but significantly lower Rab7 (Kucharava et al., 2020). Considering these findings the changes seen on the gene expression and protein level of junctional proteins could be due to decreased Rab7 presence in *Nhe6*-knockout. Thus, we speculated that disrupted endosomal signaling could have contributed to the overall downregulation of junctional gene expression, which, at the protein level, impaired their structural organization and interactions with other junctional proteins that resulted in increased permeability of endothelial layer. For example, in one study, rapid endocytosis of VE-cadherin disrupted the endothelial barrier function (Gavard and Gutkind, 2006). Additionally, the occludin C-terminal domain was crucial in paracellular channel formation and in mediating the endocytosis and trafficking of occludin (Morita et al., 1999; Ivanov et al., 2004; Schmidt et al., 2007).

We found that *Nhe6*-knockout-derived endothelial cells had significantly reduced levels of VE-cadherin and claudin-5, which are endothelial cell-specific junctional proteins (Taddei et al., 2008). Previous studies showed that a claudin-5 deletion did not affect tight junction formation, but it did result in a size-selective increase in permeability (Heiskala et al., 2001; Morganti-Kossmann et al., 2002). Both *in vitro* and *in vivo* studies have shown that VE-cadherin is a crucial component of microvascular integrity (Bazzoni et al., 2000; Nagafuchi, 2001; Ueno, 2007). In addition to the low protein level, our RNAseq assay revealed VE-cadherin as one of the most downregulated genes in *Nhe6*-knockout cells (Figure 3). VE-cadherin expression and its clustering at junctions are necessary for the transcriptional upregulation of claudin-5 (Taddei et al., 2008). It was found that NHE6 regulates anti-ICAM/NC-induced actin reorganization and subsequent internalization and vesicular trafficking by endothelial cells, which influences physiological and pathophysiological aspects, including endothelial drug delivery (Muro et al., 2006). Thus, the increased permeability in *Nhe6*-knockout cells could be primarily due to the reduced mRNA levels and disrupted vesicular trafficking of these two junctional proteins. However, the exact mechanisms underlying these effects are not completely clear.

We also found that *Slc12a2* expression and protein levels were decreased in *Nhe6*-knockout cells (Figure 4A). We mimicked this impairment in wildtype-derived endothelial cells by specifically blocking NKCC1 with a loop diuretic bumetanide. We found that this inhibition downregulated the expression of most junctional genes, except for occludin. The most prominent reductions were in the *Tjp1* and *F11r* genes, which encode ZO1 and JAM1, respectively (Figure 4B). The gene expression pattern after bumetanide treatment differed from the one found in the *Nhe*6-knockout, and this could be due to the fact that we examined only the influence of NKCC1 inhibition for 48 h and not combined effect with the lack of NHE6. When bumetanide was applied in endothelial cell and pericyte co-cultures, permeability increased (Figure 4C). NKCC1 is a Na–K-Cl cotransporter which regulates cellular osmolality, cellular volume, and fluid transport. A NKCC1 deficiency in mice caused impaired sensorineural hearing (Delpire et al., 1999; Dixon et al., 1999; Flagella et al., 1999; Diaz et al., 2007). Moreover, patients that take loop diuretics, which inhibit NKCC1, experience transient hearing loss (Rybak, 1993; Wu et al., 2014a). In many cells, an increased sodium concentration or a perturbation in cell volume leads to actin cytoskeleton reorganization and a weakening of intercellular tight junctions, which increases barrier permeability (Simard et al., 2010).

No previous study has focused on how an NHE6 deficiency might affect BLB function. Studies on blood-brain barrier dysfunction in the setting of ischemic stroke showed that an early event is the stimulation of sodium transporters (i.e., NKCC, NHE), which results in edema and the degradation of constituent TJ proteins and integrins. These events increased the paracellular leak in the barrier (Abdullahi et al., 2018). Thus, enhanced NKCC and NHE expression and activity contributed to increased Na^+^ and Cl^–^ fluxes, and subsequent water influx, through osmosis, across the barrier (Lam et al., 2009; Wallace et al., 2011). Previous reports showed that both NHE6 and NKCC1 were important for normal ion homeostasis within the cochlea. NHE6 was found to interact with the angiotensin receptor subtype 2, which mediates vasodilation. Angiotensin receptor subtype 2 is pH sensitive, pointing to the additional yet unexplored possibility of receptor function regulation by a pH microdomain created through physical interaction with NHE6 (Pulakat et al., 2005; Nagami et al., 2014). Loop diuretics are used as antihypertensives, and by unknown mechanisms, their co-administration with aminoglycosides results in greater entry of aminoglycosides into endolymph (Rybak, 1982; Taylor et al., 2008). Here, we showed that the lack or inhibition of these transporters also affected overall permeability in endothelial BLB-derived cells. Accordingly, they represent potential targets for modifying BLB permeability, which could facilitate the delivery of therapeutics across the BLB to the cochlea or protect the cochlea from ototoxic insults.

## Data Availability Statement

The datasets presented in this study can be found in online repositories. The names of the repository/repositories and accession number(s) can be found at: https://www.ncbi.nlm.nih.gov/geo/query/acc.cgi?acc=GSE196353.

## Ethics Statement

The animal study was reviewed and approved by Kantonales Veterinäramt, Basel, Switzerland.

## Author Contributions

MS-J contributed to conception and design of the study, performed the experiments, analyzed the data, and wrote the first draft of the manuscript. JP performed the experiments, organized the database, and wrote the sections of the manuscript. CS performed the measurements, data acquisition, and analysis. GA and DF contributed to sample preparation and revised the manuscript. DB provided insights and aided in interpreting the results. VP designed the figures, aided in interpreting the results, and revised the manuscript. All authors contributed to manuscript revision, read, and approved the submitted version.

## Conflict of Interest

The authors declare that the research was conducted in the absence of any commercial or financial relationships that could be construed as a potential conflict of interest.

## Publisher’s Note

All claims expressed in this article are solely those of the authors and do not necessarily represent those of their affiliated organizations, or those of the publisher, the editors and the reviewers. Any product that may be evaluated in this article, or claim that may be made by its manufacturer, is not guaranteed or endorsed by the publisher.

## Supplementary Material

Supplementary Figure 1 Cell phenotype validation: protein and gene markers are expressed in stria vascularis-derived primary endothelial cells, pericytes, and PVM/M cells. **(a)** Primary mouse stria vascularis cells were immunostained with appropriate markers for each phenotype (panels 1, 4, 6); in addition, intracellular actin was labeled with phalloidin (panels 2, 5, 8) to reveal the overall cell shape; superimposed images (merged) show intracellular localization. (Panels 1, 3) cells were stained with anti-von Willebrand factor (vWf) antibody (red); (panels 4, 6) pericytes were stained with anti-PDGFR-β antibodies (green); 1a (panels 7, 9) PVM/Ms were stained with antibodies against the macrophage marker, F4/80 (orange). **(b)** Relative mRNA levels of genes that encode cell-type markers confirm the identities of selectively cultured cell types. Endothelial cells specifically expressed *vWf*, *Slc2a1*, and *Cd34* genes; pericytes specifically expressed *Des*, *Pdgfrb*, and *Cspg4* genes. PVM/Ms specifically expressed *Gsta4* and *Adgre1* genes.

Supplementary Table 1 List of primer sequences used for qPCR.
