# Supplementary figures and images for: Lack of NHE6 and Inhibition of NKCC1 Associated With Increased Permeability in Blood Labyrinth Barrier-Derived Endothelial Cell Layer
Source: Front Cell Neurosci. 2022 Apr 12;16:862119. doi: 10.3389/fncel.2022.862119 (PMC9039518; doi:10.3389/fncel.2022.862119)

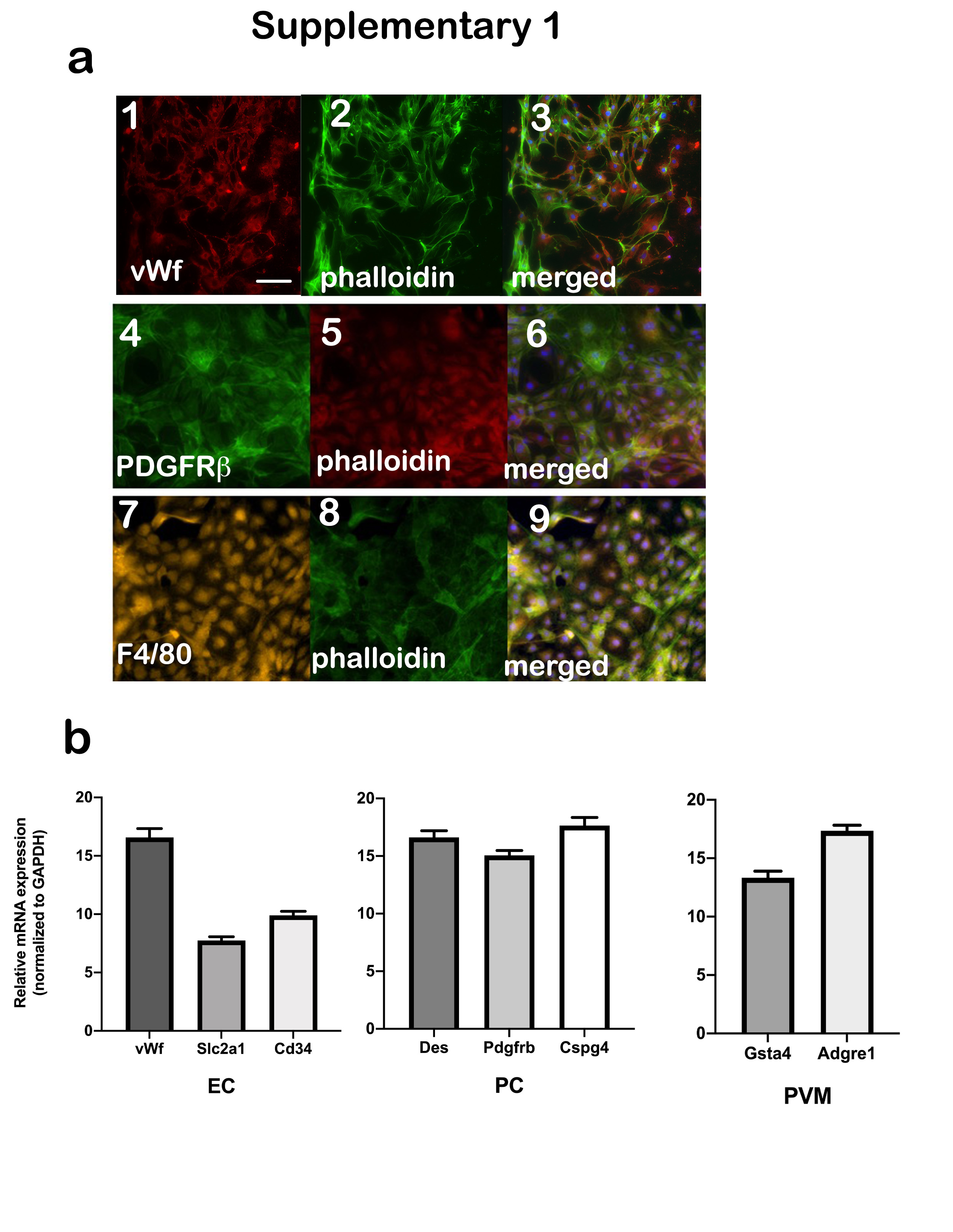

Supplement: Supplementary Figure 1 — Cell phenotype validation: protein and gene markers are expressed in stria vascularis-derived primary endothelial cells, pericytes, and PVM/M cells. (a) Primary mouse stria vascularis cells were immunostained with appropriate markers for each phenotype (panels 1, 4, 6); in addition, intracellular actin was labeled with phalloidin (panels 2, 5, 8) to reveal the overall cell shape; superimposed images (merged) show intracellular localization. (Panels 1, 3) cells were stained with anti-von Willebrand factor (vWf) antibody (red); (panels 4, 6) pericytes were stained with anti-PDGFR-β antibodies (green); 1a (panels 7, 9) PVM/Ms were stained with antibodies against the macrophage marker, F4/80 (orange). (b) Relative mRNA levels of genes that encode cell-type markers confirm the identities of selectively cultured cell types. Endothelial cells specifically expressed vWf, Slc2a1, and Cd34 genes; pericytes specifically expressed Des, Pdgfrb, and Cspg4 genes. PVM/Ms specifically expressed Gsta4 and Adgre1 genes. [file Image_1.JPEG]
