# Supplementary material for: Lack of NHE6 and Inhibition of NKCC1 Associated With Increased Permeability in Blood Labyrinth Barrier-Derived Endothelial Cell Layer
Source: Front Cell Neurosci. 2022 Apr 12;16:862119. doi: 10.3389/fncel.2022.862119 (PMC9039518; doi:10.3389/fncel.2022.862119)
Supplement: Supplementary Table 1 — List of primer sequences used for qPCR. [file Data_Sheet_1.PDF]

Primer Name	Primer sequences 5'to 3'
**vWf**	forward - CTC TTT GGG GAC GAC TTC ATC<br>reverse - TCC CGA GAA TGG AGA AGG AAC
**Slc2a1**	forward - GCA GTT CGG CTA TAA CAC TGG<br>reverse - GCG GTG GTT CCA TGT TTG ATT G
**Cd34**	forward - CTG GGT AGC TCT CTG CCT GAT<br>reverse - TGG TAG GAA CTG ATG GGG ATA TT
**Des**	forward - GTT TCA GAC TTG ACT CAG GCA G<br>reverse - TCT CGC AGG TGT AGG ACT GG
**Pdgfrb**	forward - AGG AGT GAT ACC AGC TTT AGT CC<br>reverse - CCG AGC AGG TCA GAA CAA AGG
**Cspg4**	forward - GCT GTC TGT TGA CGG AGT GTT<br>reverse - CGG CTG ATT CCC TTC AGG TAA G
**Gsta4**	forward - TAC CTC GCT GCC AAG TAC AAC<br>reverse - GAG CCA CGG CAA TCA TCA TCA
**Emr1**	forward - CTG CAC CTG TAA ACG AGG CTT<br>reverse - GCA GAC TGA GTT AGG ACC ACA A
**Tjp1**	forward - GCT TTA GCG AAC AGA AGG AGC<br>reverse - TTC ATT TTT CCG AGA CTT CAC CA
**F11r**	forward - TCT CTT CAC GTC TAT GAT CCT GG<br>reverse - TTT GAT GGA CTC GTT CTC GGG
**Ocln**	forward - TGA AAG TCC ACC TCC TTA CAG A<br>reverse - CCG GAT AAA AAG AGT ACG CTG G
**Cdh5**	forward - CAC TGC TTT GGG AGC CTT C<br>reverse - GGG GCA GCG ATT CAT TTT TCT
**Cldn5**	forward - GCA AGG TGT ATG AAT CTG TGC T<br>reverse - GTC AAG GTA ACA AAG AGT GCC A
**Gapdh**	forward - TGA CCT CAA CTA CAT GGT CTA CA<br>reverse - CTT CCC ATT CTC GGC CTT G
